# How I See and Feel About Myself: Domain-Specific Self-Concept and Self-Esteem in Autistic Adults

**DOI:** 10.3389/fpsyg.2020.00913

**Published:** 2020-05-12

**Authors:** William Nguyen, Tamara Ownsworth, Chelsea Nicol, David Zimmerman

**Affiliations:** School of Applied Psychology, Griffith University and Menzies Health Institute of Queensland, Mount Gravatt, QLD, Australia

**Keywords:** autism, self-concept, self-esteem, self-appraisals, social support, autistic adults

## Abstract

Few studies have examined the self-perceptions of autistic adults. This study aimed firstly to investigate domain-specific self-concepts and global self-esteem in autistic adults. The second aim was to examine associations between autism self-appraisals, perceived social support and global self-concept and self-esteem. The third was to determine which domains of self-concept were most closely associated with self-esteem. Participants included 71 autistic adults aged 18–70 years and 65 age, sex and education matched typically developing individuals. Participants completed an online survey of autism characteristics, global self-esteem and domain-specific self-concepts (i.e. likeability, task accomplishment, power, giftedness, invulnerability, and morality), self-appraisals about autism, and perceived social support. Autistic participants reported significantly lower power and global self-esteem than typically developing individuals after controlling for autism characteristics. More positive self-appraisals about autism (i.e. greater perceived benefits and lower helplessness) were significantly related to better global self-concept and self-esteem. Global self-esteem was significantly and positively associated with perceptions of giftedness, emotional resilience and power. These findings suggest that autistic adults may perceive themselves as having a low sense of power in their relationships and have negative global perceptions of their self-worth. However, those able to find positive meaning or benefits associated with autism are likely to have more positive global self-perceptions. This study provides new insights into how autistic adults perceive themselves which may guide the focus of psychosocial interventions that seek to recognize and promote unique talents and emotional resilience.

## Introduction

Autistic adults have been found to experience challenges in establishing their independence, forming relationships, and achieving satisfying vocational pathways (Howlin and Magiati, 2017; Moss et al., 2017). Such psychosocial difficulties may impact on their feelings of self-worth, and contribute to low self-esteem and mental health problems (Spain and Blainey, 2017). Yet, research also indicates that autistic adults often possess strengths and value certain characteristics associated with autism, such as attention to detail, excellent long-term memory and extensive knowledge of specific interest areas (Baron-Cohen et al., 1999; Markram and Markram, 2010; Lorenz and Heinitz, 2014). Currently, there is little research investigating how autistic adults perceive and feel about themselves, and the factors influencing these perceptions (Huang et al., 2017).

Self-concept refers to overall thoughts about one’s competencies, while self-esteem refers to judgments of one’s self-worth (Schweitzer et al., 1992). More positive self-concept is related to higher levels of self-esteem (Yanico and Lu, 2000); thus, these are related yet distinct constructs. Self-concept develops in a hierarchical manner with lower-order perceptions of self (e.g. context-specific self-evaluations) having a bottom-up effect on domain-specific aspects (e.g. social, work, physical, cognitive). These are subjectively weighted in terms of their influence on higher-order or global self-concept (Marsh et al., 1992). Ownsworth and Haslam (2016) highlighted that there are bi-directional influences between lower-order self-perceptions and global self-concept, such that higher-level self-representations (e.g. “I never give up”) usually inspire one’s thoughts and behavior in a specific situation, with outcomes often reinforcing global self-beliefs.

### Research on Self-Concept and Self-Esteem in ASD

In early research, Capps et al. (1995) compared the self-esteem and self-concept of autistic children without intellectual disability to typically developing (TD) peers. The autistic group had significantly lower global self-esteem and perceived social competence; however, there were no differences in perceived cognitive competence. Significant negative associations were found between autistic characteristics and social self-concept (Capps et al., 1995). Subsequent studies that employed questionnaire measures also identified that autistic children had significantly lower perceived social competence than age-based norms (Vickerstaff et al., 2007; Huang et al., 2017), and that autistic adolescents reported lower global self-esteem than TD individuals (Goddard et al., 2017).

Other studies employed the use of Damon and Hart (1988) Self-understanding Interview to assess self-conceptualization in autistic individuals (e.g. Lee and Hobson, 1998; Farley et al., 2010; Jackson et al., 2012). This interview explores seven dimensions of self-conceptualization (i.e. self-definition, self-evaluation, self in past and future, self-interest, continuity, agency and distinctiveness) that develop throughout the lifespan. In comparing the self-conceptualizations of autistic adolescents with those with intellectual disability, Lee and Hobson (1998) found that autistic adolescents reported significantly fewer social and interpersonal characteristics but were similar in levels of self-description of physical, activity and psychological characteristics. Building upon the study by Lee and Hobson (1998) and Farley et al. (2010) compared self-conceptualization from the perspective of others in autistic adolescents and typically developing controls. They found no overall differences in autistic adolescents’ ability to self-conceptualize across the dimensions (with the exception of agency, which was reduced), however, they were less able to imagine how others would view them. Farley et al. (2010) posited that only certain aspects of the self may be impaired in autism, particularly those relating to the ability to identify with others’ beliefs in relation to the self. Yet, Jackson et al. (2012) found that autistic adults (aged 19–63 years) had poorer self-understanding overall (i.e. produced fewer self-statements) compared to TD participants. Differences were significant for social, psychological and activity characteristics but not physical characteristics. Interestingly, they identified that some autistic participants were able to show better self-understanding through making a deliberate effort to better understand themselves and the world.

In a review of literature on autism and sense of self, Huang et al. (2017) proposed that autistic persons may have limited awareness of their own characteristics and differences from others due to inherent difficulties with social communication and theory of mind (ToM). Yet, they identified from reviewed studies that the self-concept of autistic children and adults was typically poorer in select areas that were related to autism characteristics (i.e. social and interpersonal domains). Global self-concept did not consistently differ from TD individuals. Such findings suggest that there is likely overlap between the characteristics of autism and self-perceptions of social competency. Hence, research investigating the self-concept of autistic individuals needs to account for the association between autism characteristics and self-perceived competencies.

Research examining factors related to self-concept in autistic participants has found that higher IQ and executive functions were associated with lower perceived social competence and global self-concept (Vickerstaff et al., 2007; Zimmerman et al., 2017). A potential explanation for these findings relates to the nature of self-appraisals, whereby heightened awareness of social difficulties and greater self-scrutiny (as supported by better reasoning, working memory and cognitive flexibility) may contribute to poorer self-concept. Qualitative studies identified that autistic adults often felt ‘different’ to their peers and experienced a sense of helplessness (Townson et al., 2007; Müller et al., 2008). Yet, they also expressed pride in their autistic identity (Hurlbutt and Chalmers, 2002), and desired acceptance from society (Müller et al., 2008). In one study, up to one-third of autistic college students did not perceive themselves as disabled or having special needs (Shattuck et al., 2014). Moreover, autistic persons who positively appraise their autistic social identity have been found to report more positive self-esteem (Cooper et al., 2017). However, further research is needed to investigate associations between self-appraisals related to autism and self-concept and self-esteem. The appraisals of interest in the current study were helplessness, acceptance and perceived benefits.

Another key appraisal that potentially influences the self-concept of autistic adults is perceived social support, or the subjective evaluation of the extent to which one’s social network is available to offer support (Kessler and McLeod, 1985). More positive perceptions of support have been found to buffer self-esteem in stressful circumstances (Cohen and Wills, 1985), and contribute to better mental health outcomes in various clinical populations (Moradkhani et al., 2013; Eadie et al., 2018), including autistic adults (Renty and Roeyers, 2006). For autistic persons, perceived availability of social support may be viewed as a positive indicator of integrating into society, thereby influencing how they perceive themselves more globally (Khanna et al., 2014). However, the relationship between perceived social support and self-concept and self-esteem in autistic adults has yet to be investigated.

In line with the preferences from most of the autism community (Kapp et al., 2013; Kenny et al., 2016), the use of identity-first language (e.g. autistic person) was adopted in this study, while acknowledging that some individuals prefer the use of person-first terminology (e.g. person with autism). Based on these preferences, more neutral terminology (i.e. autism or autistic rather than autism spectrum disorder) was used to refer to characteristics, the sample or population, in recognition that people on the autism spectrum show differences that include strengths as well as difficulties.

Interview schedules and questionnaires have previously been used to investigate the self-understanding of autistic persons (e.g. Lee and Hobson, 1998; Vickerstaff et al., 2007; Cooper et al., 2017). In the current study, the Six Factor Self-Concept Scale (SFSCS; Stake, 1994) was selected as a brief measure of domain-specific self-concepts. The SFSCS assesses interpersonal and achievement domains of self-concept, and thus potentially reflects areas of perceived competency (e.g. task accomplishment and giftedness) and low competency (e.g. socio-emotional domains of likeability and invulnerability) for the autism population.

The first aim of this study was to compare domain-specific self-concepts and global self-esteem of autistic adults to TD individuals broadly matched on age, sex and education. To extend upon previous studies, we wanted to account for the likely association between autistic characteristics and self-perceived competencies in examining between-group differences in self-concept and self-esteem. It was hypothesized that, relative to TD individuals, autistic adults would rate themselves lower on positive interpersonal attributes and have lower global self-esteem after controlling for autistic characteristics.

Although qualitative studies have highlighted that autistic adults may have both negative and positive appraisals about living with autism and their social support (Hurlbutt and Chalmers; Townson et al., 2007; Müller et al., 2008), there is a lack of research on the influence of these appraisals on their broader self-concept. Consequently, the second aim was to examine the associations between self-appraisals about autism, perceived availability of social support and global self-concept and self-esteem in autistic adults. It was hypothesized that more positive self-appraisals related to autism (i.e. greater acceptance and perceived benefits and lower helplessness) and greater perceived availability of social support would be associated with better global self-concept and self-esteem, after controlling for autism characteristics.

As previously outlined, theoretical models of self-concept (Marsh et al., 1992; Ownsworth and Haslam, 2016) propose that domain-specific self-concepts have a bottom-up influence on global self-self-esteem. However, these are subjectively weighted, with some domains having a greater influence on global self-evaluations than others. Accordingly, the third aim was to identify which domains of self-concept are most closely associated with global self-esteem in autistic adults. There was no hypothesis for this aim, thus representing an exploratory component.

## Materials and Methods

### Participants

Autistic participants were recruited via convenience sampling from Australian autism websites, support services and clinics. The inclusion criteria included: Australian residents aged >18 years who reported that they had received a formal diagnosis of autism spectrum disorder and also self-reported a history of long-term difficulties in social communication and interaction and restricted range of behaviors and/or interests as indicated by scores >77 on the Ritvo Autism Asperger’s Diagnostic Scale – Revised (RAADS-R; Ritvo et al., 2011). Eligibility was restricted to Australian residents in effort to ensure that the socio-cultural context in which participants had received a formal diagnosis of autism was similar. Although it was not possible to formally screen participants in terms of intellectual impairment or literacy difficulties, individuals with a self-reported learning disorder (e.g. dyslexia) or an acquired neurological disorder were excluded. Of the 144 adults who undertook the survey and met the RAADS-R cut-off score (>77), 96 reported a formal (professional) diagnosis of autism. Of these, 71 were Australian residents. Thus, the present sample was comprised of 71 autistic adults aged 18–70 years (*M* = 37.83, *SD* = 14.42), including 40 males (56%) and 31 females (44%). Years of education ranged from 8 to 17 (*M* = 14.31, *SD* = 2.36).

TD participants were Australian residents aged 18–70 years recruited through online social media platforms and an undergraduate psychology subject pool. TD participants with a diagnosis of autism or other neurodevelopmental disorders were excluded. TD participants were recruited after the Autistic sample, with the aim of matching the age and education characteristics and sex ratio as closely as possible at the sample level. TD participants (*n* = 65) were successfully matched to the Autistic sample on age (*t* = 0.28, *p* = 0.777), education (*t* = −0.41, *p* = 0.680) and sex (χ*^2^* = 0.22, *p* = 0.637). As shown in Table 1, the Autistic sample included a significantly higher proportion of participants with a history of psychological disorders (44.8%) than TD participants (12.3%, χ*^2^* = 16.97, *p* < 0.001). The Autistic sample also included a significantly higher proportion of participants who were unemployed or volunteering (41%) than TD participants (22%, χ*^2^* = 5.85, *p* = 0.016). Table 1 summarizes socio-demographic characteristics for both samples.

**TABLE 1:** Participants’ socio-demographic characteristics.

**Characteristics**	**Autistic sample (*n* = 71) *M*(*SD*)/*N* (%)**	**TD sample (*n* = 65) *M*(*SD*)/*N* (%)**	***t*-test/χ*^2^***	***p***	**Cohen’s *d*/Phi**
Age (years)	37.83 (14.42)	38.52 (14.03)	–0.28	0.777	0.042
**Sex**			0.22	0.637	0.040
Male	40 (56%)	34 (52.3%)			
Female	31 (44%)	31 (47.7%)			
English as a second language	3 (4.0%); 4 unknown	5 (7.7%)	0.60	0.439	0.067
Years of education	14.31 (2.36)	14.11 (3.30)	0.41	0.680	0.069
**Current occupation**					
Employed, self-employed or in higher education or vocational training	42 (59.2%)	51 (78.5%)	5.85	0.016	0.207
Unemployed or in voluntary work or job training	29 (40.8%)	14 (21.5%)			
**Relationship status**					
Not in a relationship	42 (59.0%)	37 (56.9%)	0.07	0.792	0.023
In a relationship	29 (41.0%)	28 (43.1%)			
RAADS-R	149.30 (32.65)	56.57 (39.13)	15.05	< 0.001	2.57
**Psychological history^a^**			16.97	< 0.001	0.359
No	37 (52.1%)	57 (87.7%)			
Yes	30 (42.3%)	8 (12.3%)			
Unknown	4 (5.6%)	−			

*^*a*^Participants were asked to indicate whether they had a history of a psychological disorder (i.e. Do you have a history of a major psychiatric disorder? [e.g. major depressive disorder, generalized anxiety disorder, schizophrenia]); RAADS-R = Ritvo Autism Asperger’s Diagnostic Scale – Revised.*

### Measures

#### Ritvo Autism Asperger’s Diagnostic Scale-Revised

The RAADS-R (Ritvo et al., 2011) is a self-report measure based on the DSM-IV-TR (American Psychiatric Association, 2000) and ICD-10 (World Health Organization, 1992) criteria to assist in the diagnosis of autism. The 80-item measure is comprised of four sub-scales: Social Relatedness (e.g. “I am a sympathetic person”), Circumscribed Interests (e.g. “I focus on details rather than the overall idea”), Sensory Motor (e.g. “Some ordinary textures that do not bother others feel very offensive when they touch my skin”), and Social Anxiety (e.g. “It can be very intimidating for me to talk to more than one person at the same time”). Items are rated on a 4-point Likert scale (0 = True now and when I was young, to 3 = Never true) with higher scores indicating greater autism characteristics. The RAADS-R has good to excellent internal consistency (α = 0.87–0.95) and excellent test-retest reliability (*r* = 0.99) over a 1-year period. A cut-off score of >77 has been found to have good sensitivity (>0.90) and specificity (>0.87) (Andersen et al., 2011). Internal consistency was excellent for the current Autistic (α = 0.91) and TD (α = 0.95) samples.

#### Rosenberg Self-Esteem Scale

The RSES (Rosenberg, 1965) is a measure of global self-esteem consisting of 10-items scored on a 4-point Likert scale (3 = strongly agree, to 0 = strongly disagree). Five items are positively worded and five are negatively worded (reverse scored). The items reflect global judgments of one’s self-worth rather than perceptions related to a particular domain or set of attributes. An example positively worded item includes “I feel that I have a number of good qualities”. An example of a negatively worded item includes “I feel I do not have much to be proud of”. Higher scores indicate more positive self-esteem. The RSES displays excellent internal consistency (α = 0.94; Zimmerman et al., 2017) and good test-retest reliability over a 4 week period (*r* = 0.84; Martin-Albo et al., 2007). There is evidence of concurrent validity with measures of mood in autistic adults (*r* = −0.55, *p* < 0.001; Zimmerman et al., 2017). Internal consistency was excellent for the Autistic (α = 0.92) and TD (α = 0.90) samples.

#### Six-Factor Self-Concept Scale for Adults

The SFSCS (Stake, 1994) is a 36-item multi-domain self-concept measure comprised of six factor-derived subscales: likeability (e.g. sociable), morality (e.g. honest), task accomplishment (e.g. productive), giftedness (e.g. special talents), power (e.g. dominant), and vulnerability (e.g. lacks confidence). Items are rated on a Likert scale (1 = never or almost never true, to 7 = always or almost always true). Unlike the RSES, the SFSCS items relate to self-perceptions of specific psychological attributes (e.g. honest, warm), with higher scores indicating more positive domain-specific self-concept. The total or sum of scores across the domains reflects global self-concept. Scores on the vulnerability subscale are reversed to reflect ‘invulnerability’ (i.e. emotional resilience). Internal consistency (α = 0.74–0.89) and test-retest reliability are found to range from acceptable to good (*r* = 0.74–0.88; Stake, 1994). The SFSCS was found to be highly correlated with the RSES in autistic adults (*r* = 0.62–0.67, *p* < 0.001; Yanico and Lu, 2000; Zimmerman et al., 2017). In this study, internal consistency of the total score was good (Autistic α = 0.87; TD α = 0.91) and ranged from satisfactory to good for the subscales (α = 0.79–0.88).

#### Autism Self-Appraisals Questionnaire

Given the lack of validated tools for assessing cognitive appraisals related to autism, we adapted the Illness Cognitions Questionnaire (ICQ; Evers et al., 2001), a validated self-report measure developed to assess self-appraisals of helplessness, acceptance and perceived benefits associated with symptoms of a chronic health condition (Lauwerier et al., 2010). The instructions and 18-items were modified to refer to appraisals about having autism whereby participants were asked to indicate the extent to which they agreed with each item. Example items for each subscale were as follows: helplessness ([having autism] “frequently makes me feel helpless” and “prevents me from doing what I would really like to do”; acceptance (“I can handle the problems related to” [having autism] and “I have learned to live with” [having autism]; and perceived benefits ([having autism] “has made me a stronger person” and “I have learned a great deal from” [having autism]). Each scale is comprised of six items rated on a 4-point Likert scale (1 = not at all, to 4 = completely), with higher scores indicating greater appraisals of helplessness, acceptance and perceived benefits. Internal consistency for the three scales was good (α = 0.84–0.88) for the current Autistic sample.

#### Interpersonal Support Evaluation List-Short Form

The ISEL-SF (Cohen and Hoberman, 1983) is a 16-item measure of perceived social support that is comprised of four sub-scales (i.e. appraisal, tangible assets, belonging, and self-esteem support) (Brookings and Bolton, 1988). Example items for each subscale were as follows: appraisal (e.g. “When I need suggestions on how to deal with a personal problem, I know someone I can turn to”), tangible assets (e.g. “It would be difficult to find someone who would lend me their car for a few hours”), belonging (e.g. “I don’t often get invited to do things with others”), and self-esteem (e.g. “Most of my friends are more successful at making changes in their lives than I am”) support. Items are scored on a 4-point scale (0 = definitely false, to 3 = definitely true, with higher overall scores representing greater perceived availability of social support from their own perspective. The ISEL-SF has been used previously in validation research for autistic people (see McConachie et al., 2018), and demonstrates good internal consistency for the total score (α = 0.83), and convergent validity with measures of social support (Emery et al., 2004; Payne et al., 2012). Internal consistency of the ISEL-SF total score was good (α = 0.83) for the current Autistic sample.

### Procedure

Ethical clearance was obtained from the Griffith University Human Research Ethics Committee (protocol number PSY/28/13/HREC) and the study was conducted in accordance with the National Statement on Ethical Conduct in Human Research. A flyer advertising the study was sent to the coordinators of Australian autism support services and clinics who distributed the survey link via their website or provided hard copies of the flyer. Participants opened the link which took them to the consent form and a web-based survey platform (LimeSurvey) that was used to administer the self-report measures. An information sheet and consent mechanism was initially presented, followed by the socio-demographic and health background survey, RAADS-R, RSES, SFSCS, ASQ, and ISEL-SF. TD participants were recruited via the social networks of the researchers using online social media platforms and an undergraduate psychology subject pool. After expressing interest in the study they were sent an email with the survey link. They completed the same measures as the autistic sample with the exception of the ASQ and ISEL-SF.

### Data Analysis

Data analyses were conducted using the Statistical Package for the Social Sciences, Version 25. The data were screened and managed for missing values and relevant parametric assumptions. Missing data on the SFSCS (*n* = 3), and ASQ (*n* = 3) for Autistic participants was managed using pairwise exclusion, analysis-by-analysis. In relation to the first aim, MANCOVA and ANCOVA were used to investigate group differences in domain-specific self-concept (SFSCS) and global self-esteem (RSES), controlling for relevant covariates. For the second aim, preliminary Pearson’s correlations were conducted to identify relevant covariates and regression analyses were then conducted to investigate associations between autism self-appraisals (ASQ), perceived availability of social support (ISEL-SF) and global self-concept and self-esteem. For the exploratory component, a hierarchical regression was employed to examine associations between the SFSCS domains and RSES, controlling for RAADS-R. Two-tailed significance tests (*p* < 0.05) were used for all analyses.

## Results

### Comparison of Self-Concept and Self-Esteem Between the Autistic and TD Groups

The assumptions for conducting ANCOVA (RSES) and MANCOVA (SFSCS) were satisfied, with two exceptions. First, homogeneity of regression slopes was violated for the SFSCS morality domain, indicating that the relationship between the covariate (RAADS-R) and morality differed for the two groups. In line with recommendations of Tabachnick and Fidell (2019), the morality dependent variable was omitted from the MANCOVA. Second, homogeneity of variance was violated for the SFSCS domain Likeability; consequently, a more stringent alpha level was adopted, as outlined below. In terms of potential covariates, there were no significant associations between RAADS-R or psychological history and task accomplishment, power, giftedness and morality (*r* = 0.05–0.15, *p* = 0.074–0.588). RAADS-R, but not psychological history, was significantly correlated with RSES (*r* = −0.37, *p* < 0.001). Both RAADS-R (*r* = −0.46–0.62, *p* < 0.001) and psychological history (*r* = 0.21–0.26, *p* = 0.016) were related to invulnerability and likeability; however, psychological history was no longer significantly related to likeability and invulnerability when controlling for total RAADS-R (partial *r* = −0.01–0.02). Further, there were no differences on the self-concept domains for individuals who were employed or in higher education as compared to those who were not employed or in higher education (all *p*-values < 0.10). Therefore, only RAADS-R was treated as a covariate in both the MANCOVA (SFSCS) and ANCOVA (RSES).

A one-way MANCOVA controlling for autism characteristics revealed a significant overall effect of group on the self-concept domains, Wilk’s λ = 0.91, *p* = 0.042 (see Table 2). Univariate comparisons were examined for each self-concept domain using ANCOVA with a Bonferroni correction for multiple comparisons (α = 0.05/5 = 0.01). To account for the violation of homogeneity of variance, the self-concept domain Likeability had an adjusted *p*-value of.005 (0.01/2). As shown in Table 2, there was only one significant between-group difference, which was on the power domain (*p* = 0.007, η*_*p*_^2^* = 0.05), with autistic persons reporting lower power in relationships than TD participants. Further, a one-way ANCOVA controlling for autism characteristics (*F* = 19.67, *p* < 0.001, η*_*p*_^2^* = 0.13) identified that autistic participants reported significantly lower global self-esteem on the RSES (*M* = 16.60, *SD* = 7.72) than TD participants (*M* = 19.32, *SD* = 5.85), *F*(1, 133) = 4.08, *p* = 0.045, η*_*p*_^2^* = 0.03. An independent *t*-test was conducted for the morality domain, which indicated no significant difference in scores between the Autistic (*M* = 37.51, *SD* = 4.29) and TD groups (*M* = 36.70, *SD* = 5.11; *t*(131) = 1.01, *p* = 0.316, *d* = 0.17). In summary, autistic participants had a lower sense of power in relationships and poorer self-esteem than TD participants, after controlling for autism characteristics.

**TABLE 2:** MANCOVA and Univariate Pairwise Comparisons of the Six Factor Self Concept Scale (SFSCS) between the Autistic and TD Groups (note: EMM = Estimated Marginal Means, adjusted for RAADS-R).

**Variables**	**Autistic (*n* = 68)**		**TD (*n* = 65)**		**Wilk’s λ**	***F***	***p*^a^**	**$\eta_{p}^{2}$**
	***EMM***	***SE***	***EMM***	***SE***				
MANCOVA Result					0.91	2.38	0.042*	0.09
RAADS-R (covariate)					0.61	16.48	< 0.001***	0.39
**SFSCS**								
Likeability	29.45	0.99	30.28	1.02		0.24	0.626^a^	0.00
Task Accomplishment	31.00	1.06	32.95	1.10		1.12	0.291	0.01
Power	25.71	1.24	31.58	1.28		7.47	0.007**	0.05
Giftedness	22.92	1.08	22.70	1.11		0.02	0.904	0.00
Invulnerability	22.47	1.09	19.53	1.13		2.41	0.123	0.02

*p < 0.05, **p < 0.01, ***p < 0.001; ^*a*^Likeability had an adjusted *p*-value of.005 (0.01/2) to account for the violation of homogeneity of variance.

### Associations Between Autism Appraisals, Social Support and Self-Concept and Self-Esteem

Preliminary correlation analyses were conducted to identify potential covariates (i.e. socio-demographic and autism characteristics) for the regression analysis involving global self-concept (SFSCS) and self-esteem (RSES). As shown in Table 3, there were no significant associations between age, education and employment status and global self-concept or self-esteem. There was a tendency for males to report higher self-esteem than females (*r* = 0.23, *p* = 0.051), although this did not reach significance. Autism characteristics were negatively associated with self-esteem (*r* = −0.27, *p* = 0.023), but were not significantly related to global self-concept (*r* = 0.004, *p* = 0.975). Greater acceptance (*r* = 0.32, *p* = 0.009) and perceived benefits (*r* = 0.56, *p* < 0.001) of autism were significantly associated with better global self-concept. However, there was no significant association between helplessness and global self-concept (*r* = −0.11, *p* = 0.372). Further, greater acceptance (*r* = 0.57, *p* < 0.001) and perceived benefits (*r* = 0.58, *p* < 0.001), and lower helplessness (*r* = −0.47, *p* < 0.001) were significantly associated with higher self-esteem. Perceived social support was significantly and positively associated with global self-concept (*r* = 0.33, *p* = 0.008) and self-esteem (*r* = 0.54, *p* < 0.001).

**TABLE 3:** Descriptive statistics and correlations between demographic variables, autism traits, self-appraisals, perceived social support, global self-concept and self-esteem for autistic participants.

	**Helplessness**	**Acceptance**	**Perceived benefits**	**Perceived social support**	**Global self-concept**	**Self-esteem**
**Descriptive statistics**						
*M (SD)*	13.03 (4.11)	16.03 (3.40)	15.94 (4.80)	23.48 (9.03)	164.15 (23.81)	16.61 (7.72)
Age	–0.12	0.09	–0.02	−0.27*	0.09	0.15
Sex^*a*^	–0.09	0.23	–0.004	0.22	0.04	0.23*
Education	–0.05	0.15	0.03	0.02	0.12	0.11
Employment status^*b*^	–0.17	0.04	0.01	0.09	0.11	0.05
RAADS-R	0.42***	–0.10	0.06	−0.27*	0.004	−0.27*
**Autism self-appraisals**						
Helplessness	−	−0.31*	–0.13	−0.43***	–0.11	−0.47***
Acceptance	−0.31*	−	0.58***	0.34**	0.32**	0.57***
Perceived Benefits	–0.13	0.58***	−	0.42***	0.56***	0.58***
Perceived social support	−0.43***	0.34**	0.42***	−	0.33**	0.54***

*p < 0.05, **p < 0.01, ***p < 0.001; ^*a*^1 = female, 2 = male; ^*b*^0 = not employed/studying, 1 = employed or studying.

A hierarchical regression analysis was conducted to determine whether autism self-appraisals and perceived social support were related to global self-esteem after controlling for autism characteristics and sex (see Table 4). In step 1, autism characteristics and sex significantly contributed to variance in self-esteem, *R*^2^ = 0.09, adjusted *R*^2^ = 0.06, *F*(2, 65) = 3.17, *p* = 0.048. When autism self-appraisals (acceptance, perceived benefits and helplessness) and perceived social support were entered in Step 2, the total variance explained increased to 57.7%, *R*^2^ = 0.58, adjusted *R*^2^ = 0.54, *F*(6, 61) = 13.89, *p* < 0.001. Autism self-appraisals and perceived social support accounted for an additional 49% of variance in self-esteem (Δ*R*^2^ = 0.49; Δ*F*(4, 61) = 17.63; *p* < 0.001). Helplessness (β = -0.24, *p* = 0.024) and perceived benefits (β = 0.37, *p* = 0.002) accounted for significant unique variance (*sr^2^* = 0.04–0.07) in the model.

**TABLE 4:** Hierarchical regression of autism characteristics (raads-r), sex, autism self-appraisals and perceived social support on global self-esteem.

**Variables**	***R*^2^**	****Δ***R*^2^**	***B***	***SE (B)***	**β**	***t***	***sr*^2^**
Step 1	0.11*	0.11*					
RAADS-R			–0.05	0.03	–0.24	–2.02	0.05
Sex			2.99	1.83	0.19	1.64	0.02
Step 2	0.58***	0.48***					
RAADS-R			–0.02	0.02	–0.10	–1.04	0.01
Sex			1.10	1.42	0.07	0.77	0.00
Helplessness			–0.45	0.19	–0.24	−2.35*	0.04
Acceptance			0.43	0.25	0.19	1.71	0.02
Perceived benefits			0.63	0.18	0.39	3.46**	0.07
Perceived social support			0.13	0.09	0.15	1.42	0.02

*p < 0.05, **p < 0.01, ***p < 0.001; RAADS-R = Ritvo Autism Asperger’s Diagnostic Scale–Revised.

For global self-concept, a standard multiple regression was conducted with the autism self-appraisals of helplessness, acceptance and perceived benefits and perceived social support. A significant overall effect was found, *R*^2^ = 0.32, adjusted *R*^2^ = 0.28, *F*(4,63) = 7.55, *p* < 0.001. As shown in Table 5, only perceived benefits accounted for significant unique variance in global self-concept (β = 0.52, *p* < 0.001, *sr*^2^ = 0.16). Therefore, perceiving greater benefits associated with autism was related to higher self-esteem and more positive global self-concept.

**TABLE 5:** Standard regression of helplessness, acceptance, perceived benefits and perceived social support on global self-concept.

**Variables**	***R*^2^**	****Δ***R*^2^**	***B***	***SE (B)***	**β**	***t***	***sr*^2^**
Overall model	0.32	0.32***					
Helplessness			0.01	0.69	0.002	0.02	0.00
Acceptance			–0.17	0.93	–0.02	–0.18	0.00
Perceived benefits			2.60	0.67	0.52	3.98***	0.16
Perceived social support			0.31	0.33	0.12	0.95	0.01

***p < 0.001.

**TABLE 6:** Hierarchical regression of the self-concept domains on global self-esteem for the autistic sample, controlling for autism characteristics.

**Variables**	***R*^**2**^**	****Δ***R*^**2**^**	***B***	***SE (B)***	**β**	***t***	***sr*^**2**^**
Step 1	0.07*	0.07*					
RAADS-R			–0.06	0.03	–0.26	−2.23*	0.07
Step 2	0.55***	0.48***					
RAADS-R			–0.07	0.03	–0.31	−2.78**	0.06
Likeability			0.01	0.11	0.01	0.06	0.00
Task Accomplishment			0.06	0.13	0.05	0.45	0.00
Power			0.20	0.10	0.20	2.05*	0.03
Giftedness			0.40	0.12	0.36	3.28**	0.08
Invulnerability			0.35	0.12	0.33	3.03**	0.07
Morality			0.30	0.20	0.17	1.51	0.01

*p < 0.05, **p < 0.01; RAADS-R = Ritvo autism asperger’s diagnostic scale–Revised.

### Associations Between Self-Concept Domains and Global Self-Esteem

A hierarchical regression analysis was conducted to identify the domains of self-concept most closely associated with global self-esteem. After controlling for autism characteristics in Step 1 [*R*^2^ = 0.07, adjusted *R*^2^ = 0.06, *F*(1, 66) = 0.06, *p* = 0.029], giftedness (β = 0.36, *p* = 0.002), invulnerability (β = 0.33, *p* = 0.004) and power (β = 0.20, *p* = 0.045) each accounted for unique variance in self-esteem in Step 2 [Δ*R*^2^ = 0.48, Δ*F*(6, 60) = 10.54, *p* < 0.001]. Likeability (β = 0.01, *p* = 0.956), task accomplishment (β = 0.05, *p* = 0.658), and morality (β = 0.17, *p* = 0.137) did not account for significant variance in Step 2 of the model. Therefore, greater self-perceived giftedness, invulnerability and power were independently associated with higher global self-esteem (*sr^2^* = 0.03–0.08), controlling for autism characteristics.

## Discussion

This study aimed to investigate self-concept and self-esteem in autistic adults, and associations with self-appraisals related to autism and perceived social support. A key finding was that autistic adults reported a lower sense of power in relationships and poorer global self-esteem than TD individuals after controlling for autism characteristics. In contrast to the hypothesis, there were no significant differences on the domains of likeability, task accomplishment, giftedness, invulnerability and morality. More positive autism self-appraisals and perceptions of social support were related to better global self-concept and higher self-esteem. In the multivariate analyses, after controlling for autism characteristics and sex, greater perceived benefits and lower helplessness were significantly associated with higher self-esteem. For the third exploratory aim, greater perceptions of giftedness, invulnerability and power were associated with higher self-esteem after controlling for autism characteristics.

As measured by the SFSCS, power refers to the perceived ability to influence others, and relates to characteristics of dominance, leadership, and aggressiveness (Stake, 1994). The combined attributes of the power domain suggests the presence of extraverted qualities in relationships (e.g. assertiveness), which could aid in goal-attainment (Flood et al., 2011). Conversely, attributes such as aggressiveness can be maladaptive and result in peer rejection (Snyder et al., 2004). Given the social-communication challenges often experienced by autistic persons, it is perhaps unsurprising that they experience lower self-concept in this domain. For instance, autistic persons who report low assertiveness skills have been found to report higher levels of social anxiety (Bellini, 2004; Turner and Hammond, 2016). Furthermore, interventions to improve assertiveness skills in autistic persons have had mixed success (Howlin and Yates, 1999; Gantman et al., 2012). In a qualitative study, autistic persons expressed reservations about standing up for themselves due to concern that they would offend other people (Haertl et al., 2013; Howlin and Yates, 1999). Howlin and Yates (1999) described one autistic adult as having been financially manipulated by a ‘friend’, and lacked assertiveness upon repeat encounters despite acknowledging that he had been manipulated. Further research is needed to examine the influence of perceived power in social situations for autistic adults.

Extending upon previous research (Capps et al., 1995; Goddard et al., 2017; McCauley et al., 2017), the current study identified that autistic adults experience lower self-esteem than TD individuals after controlling for the association between autism characteristics and self-esteem. Therefore, their negative global self-evaluations cannot be attributed to the extent of their autism characteristics. Longitudinal studies have shown that global self-esteem is relatively stable across the life course and that high self-esteem is predictive of positive outcomes in multiple life domains (e.g. better relationships, work, and health), even after accounting for earlier levels of self-esteem and life success (Crocker and Wolfe, 2001; Orth and Robins, 2014). This is potentially because those with high levels of self-esteem tend to have greater persistence on challenging tasks and have more effective self-regulation strategies (Di Paula and Campbell, 2002). Accordingly, autistic persons who report low self-esteem may experience greater difficulties in persisting with goals perceived as challenging (e.g. employment, social relationships). For instance, autistic persons have reported feeling dejected at being under-employed relative to their capabilities (Carpenter, 1992). Further, Haertl et al. (2013) found that autistic adults often felt discouraged by previous attempts at managing social situations (e.g. “I didn’t try to interact with other students because I didn’t know how…It felt too complicated when I tried”; p. 33), which may contribute to the experience of loneliness and perpetuate their low self-esteem (Mazurek, 2014).

Contrary to the hypothesis, autistic participants did not report poorer self-concept in the domains of likeability or invulnerability compared to TD participants, after controlling for autism characteristics. Notably, these self-concept domains were significantly correlated with autism characteristics on the RAADS-R (*r* = −0.46–0.62, *p* < 0.001). As measured by the SFSCS, likeability refers to the interpersonal qualities of friendliness, warmth and sociability, whereas invulnerability refers to emotional resilience (e.g. easily hurt, self-conscious). It is likely that likeability and invulnerability attributes are closely tied to the social relatedness and social anxiety characteristics of autism (e.g. “only like to talk to people who share my special interests” “Meeting new people is usually easy for me” [reverse coded]). These findings highlight the importance of distinguishing the characteristics of autism from self-concept in related socio-emotional domains.

In general, more positive autism self-appraisals and perceptions of social support were significantly correlated with higher self-esteem and better global self-concept. However, the self-appraisal of acceptance was not independently related to global self-esteem or self-concept. Acceptance self-appraisals relate to learning to live with, handling problems or accepting limitations (Evers et al., 2001) associated with autism. Although endorsing these items may reflect active coping efforts, it is possible that acceptance *per se* may not promote positive reinterpretation of autism or enhance global feelings of self-worth. Further, greater perceptions of helplessness were independently related to more negative judgments of one’s self-worth, but not global self-concept. This may be because self-appraisals of helplessness focus on feelings about having autism (e.g. helpless and useless) rather than overall perceptions of self-competency. More positive perceptions of social support were significantly related to higher self-esteem and global self-concept, although this was no longer the case when autism characteristics and other autism self-appraisals were accounted for. As supported by the significant negative association between perceived social support and autism characteristics (*r* = −0.27), it is plausible that perceptions of social supports are closely tied to social communication characteristics of autism; such as the perception that there are several people to talk to when a person feels lonely (Cohen and Wills, 1985).

Although the autism self-appraisals were significantly interrelated (*r* = −0.31–0.58), only perceived benefits accounted for unique variance in the multivariate models for both self-esteem and self-concept. These findings broadly align with previous research on benefit finding and psychological well-being in the context of life stressors. Specifically, perceiving benefits or finding positive meaning in living with a chronic health condition has been shown to have long-term positive effects on physical and psychological health (Affleck et al., 1987; Evers et al., 2001). For autistic persons, the ability to positively appraise what it means to live on the spectrum may be adaptive for self-worth through recognizing personal strengths. In line with the neurodiversity movement, autism is increasingly viewed as a way of being, rather than a health condition (Kapp et al., 2013). Instead of promoting neurotypical functioning, neurodiversity advocates encourage individuals to embrace their strengths and differences associated with autism in order to promote well-being and self-actualization (Ne’eman, 2010; Robertson, 2010). Given that children construct a working model of the self from a young age (Harter, 2012), the current findings highlight the value of caregivers supporting individuals to find positive meaning in being on the spectrum and to discover their particular talents and strengths (Haertl et al., 2013).

Overall, this study demonstrated that autistic adults perceive a lower sense of power and poorer global self-esteem. Further, after controlling for autism characteristics, there were significant positive associations between the giftedness, invulnerability and power domains and global self-esteem. As measured by the SFSCS, giftedness refers to natural aptitudes, special talents and ingenious qualities. Consistent with theoretical models of self-concept (Marsh et al., 1992), domain-specific perceptions of talents or gifts in certain areas (e.g. sporting, artistic, musical) may engender a strong sense of pride, that enhances global perceptions of self-worth (Smith and Sharp, 2013). Alternatively, more positive global self-evaluations (e.g. I can master skills with effort) could motivate individuals to approach and persist with new challenges, such as pursuing hobbies or careers that foster personal strengths (Lindstrom et al., 2013; Ownsworth and Haslam, 2016). Similarly, perceptions of emotional resilience (i.e. invulnerability) and power in relationships (i.e. perceived ability to influence others) may protect from threats to self-esteem and support individuals to develop effective ways of coping with challenging life situations (Flood et al., 2011).

### Study Limitations

Several limitations of this study are important to acknowledge, particularly in relation to the convenience sample and cross-sectional design. Due to potential self-selection bias and the high proportion of females (44%) relative to population-based estimates (20%; Werling and Geschwind, 2013), the current sample may not be broadly representative of the autistic adult population. Participants were recruited via Australian autism websites and services which provide information and support, and thus they may have more favorable attitudes about help seeking and autism research than non-participants. Additionally, the use of a cross-sectional design means that conclusions cannot be drawn regarding the direction of associations between autism characteristics, appraisal variables and self-esteem and self-concept. In future research it is recommended that a longitudinal study be conducted with a more representative sample to investigate how autism self-appraisals influence, and are influenced by global self-perceptions across the life course for autistic adults.

A further limitation relates to the lack of formal verification of a current autism diagnosis from an independent clinical assessment. Nevertheless, all participants reported longstanding difficulties in social communication skills and a restricted range of behaviors and/or interests on the RAADS-R. As noted by Lai et al. (2015), there may be autism-specific sex differences, and it is unclear whether the RAADS-R is sensitive to autistic traits in the female population. More generally, the RAADS-R is based on the DSM-IV-TR (American Psychiatric Association, 2000) rather than the DSM-5 (American Psychiatric Association, 2013). It is possible that some items on this measure are outdated and not consistent with the latest diagnostic criteria, which consolidates three previous categories (social impairment, language and communication impairment and repetitive/restricted behaviors) of autism symptoms into two categories and the inclusion of sensory issues under the restricted/repetitive behavior category. Additionally, due to the administration of self-report measures via the internet, it was not possible to screen participants in terms of intellectual impairment or literacy difficulties, which may have impacted on their ability to independently complete questionnaires and ratings of self-concept. Although level of education was not significantly associated with self-concept or self-esteem (see Table 3), the impact of intellectual functioning and language ability on participants’ self-perceptions could not be determined. A formal screening for intellectual impairment and independent verification of participants’ diagnosis of autism based upon DSM-5 criteria is recommended in future research.

Further, due to the focus on individuals’ perceptions of themselves and their social support, only self-report measures were employed. In relation to social support, the ISEL-SF does not account for individuals’ social cognition or awareness of their own social support needs, or values and preferences regarding social support (e.g. preference for larger and more extensive network vs. a smaller and more intimate support network). It is also important to acknowledge the potential influence of method variance on the findings. More specifically, it is possible that common response formats (e.g. self-report Likert scales) artificially inflated the covariance between these measures. However, as noted by Spector (2006), if method variance is a major issue, significant correlations should be observed among all variables assessed using the same method, which was not the case in this study.

In terms of future research, the current findings highlight the need to investigate factors influencing autism self-appraisals and to identify ways of supporting autistic adults to derive positive meaning from living on the spectrum. For example, understanding the influence of social environmental factors (e.g. family dynamics, peer support, educational and vocational opportunities) on self-appraisals could guide the focus of psychosocial interventions and initiatives to promote personal talents and strengths (Hurlbutt and Chalmers, 2002; Shattuck et al., 2014). Such research would ideally be informed by and developed with autistic individuals in accordance with co-design principles (Francis et al., 2009).

## Conclusion

While previous studies of self-concept and self-esteem have reported that autistic adolescents have lower self-worth and perceived social competence relative to TD participants (Capps et al., 1995; Huang et al., 2017), there has been limited research on domain-specific self-concept and global self-esteem in autistic adults. This study compared the domain-specific self-concepts and self-esteem of autistic adults with TD individuals, and examined associations between autism self-appraisals, perceived social support and self-concept and self-esteem. The key findings were that autistic adults reported a lower sense of power in relationships and poorer global self-esteem than TD individuals, and that more positive self-appraisals of autism and perceptions of social support were related to better global self-concept and higher self-esteem. Importantly, those who perceived greater benefits associated with autism were found to have more positive global self-concept and higher self-esteem. Further, adults who see themselves as having special talents, power in relationships and emotional resilience had greater self-worth irrespective of their autism characteristics. Overall, this study provides new insights into how autistic adults perceive and feel about themselves, which may guide the focus of psychosocial interventions. It is recommended that future research investigate factors influencing autism self-appraisals and develop interventions that support individuals to recognize the benefits of autism and foster their unique talents and strengths.

## Data Availability Statement

The datasets generated for this study are available on request to the corresponding author.

## Ethics Statement

The studies involving human participants were reviewed and approved by the Griffith University Human Research Ethics Committee (protocol number PSY/28/13/HREC). The patients/participants provided their written informed consent to participate in this study.

## Author Contributions

All authors made substantial contributions to the conception and design of the study and agreed to be accountable for all aspects of the work in ensuring that questions related to the accuracy or integrity of any part of the work are appropriately investigated and resolved. WN, CN, and DZ were involved in the recruitment of participants, the data collection, and data entry. WN and TO were involved in the data analysis and were responsible for preparing initial drafts of the manuscript. Each author was involved in drafting the work and/or critically revising it for important intellectual content and all authors gave final approval of the version to be published.

## Conflict of Interest

The authors declare that the research was conducted in the absence of any commercial or financial relationships that could be construed as a potential conflict of interest.
